# BDNF gene polymorphysm RS2049046 in episodic and chronic migraine

**DOI:** 10.1186/1129-2377-14-S1-P16

**Published:** 2013-02-21

**Authors:** Y Azimova, A Sergeev, K Skorobogatykh

**Affiliations:** 1 1st Moscow State Medical University, Russian Federation

## Background

The brain-derived neurotrophic factor (BDNF) is closely involved in the pathophysiology of mood disorders and pain processing. An increased risk for the AT-genotype of rs2049046 in BDNF gene and the GC-genotype of rs1553005 in CGRP gene was previously reported in migraine patients.

## Aim

The aim of the study was to investigate the prevalence of BDNF gene polymorphism rs2049046 in chronic and episodic migraine.

## Patients and methods

117 patients with migraine (ICHD II), 12 men and 105 women, mean age 40.7±12.1 y.o. were included. 78 patients had episodic migraine (EM) and 39 had chronic migraine (CM). SNP rs2049046 was genotyped by PCR-RLFP technique: PCR with “GenPakTM PCR Core” (Isogene Laboratory, Ltd) and restriction with HinfI (SibEnzyme Ltd).

## Results

The prevalence of TT genotype of rs2049046 was significantly higher in CM group (20.5%) compared with EM group (6.4%), p=0.022. The prevalence of AA (8.9% in EM group and 7.7% in CM group) and AT genotypes (84.6% in EM group and 71.8% in CM group) did not differ significantly.

## Conclusions

TT genotype of rs2049046 in BDNF gene appears to influence susceptibility to migraine chronification. This polymorphism could also be a link for comorbidity of chronic migraine and mood disorders.
